# Imagining the future of optical microscopy: everything, everywhere, all at once

**DOI:** 10.1038/s42003-023-05468-9

**Published:** 2023-10-28

**Authors:** Harikrushnan Balasubramanian, Chad M. Hobson, Teng-Leong Chew, Jesse S. Aaron

**Affiliations:** Advanced Imaging Center; Howard Hughes Medical Institute Janelia Research Campus, Ashburn, VA 20147 USA

**Keywords:** Imaging, Microscopy

## Abstract

The optical microscope has revolutionized biology since at least the 17^th^ Century. Since then, it has progressed from a largely observational tool to a powerful bioanalytical platform. However, realizing its full potential to study live specimens is hindered by a daunting array of technical challenges. Here, we delve into the current state of live imaging to explore the barriers that must be overcome and the possibilities that lie ahead. We venture to envision a future where we can visualize and study everything, everywhere, all at once – from the intricate inner workings of a single cell to the dynamic interplay across entire organisms, and a world where scientists could access the necessary microscopy technologies anywhere.

## Abstract (web summary)

A high-level perspective on the future of live imaging is presented, with a vision to image anything in a biological sample, anywhere and at any time. A particular focus is given to current challenges and strategies to overcome them.

## Introduction

Optical microscopy remains one of the most rapidly developing technologies in scientific research^1–3^. Compared to other techniques in life science, optical microscopy makes it possible for us to visualize biology in its physiological context. In a short span of less than three decades, several important breakthroughs in light microscopy have revolutionized life sciences. These include, but are not limited to: (i) genetically encoded fluorescent proteins for live cell imaging^4–6^, (ii) light sheet microscopy^7–12^, (iii) super-resolution microscopy^13–24^, (iv) label-free imaging approaches^25–29^, (v) machine learning^30–34^, and (vi) imaging technologies capable of adapting to the biology of the specimens^35–46^. Together, these innovations have allowed biologists to understand the fundamental processes of life across a large range of spatiotemporal scales or under conditions previously considered incompatible with imaging.

Despite these achievements, we continue to face obstacles in deciphering the interplay among the many processes that together sustain life. This is due largely to the limited capability of current technologies to combine all the essential imaging parameters required to comprehend the totality of the biology in question. For instance, it is extraordinarily challenging and often impossible to simultaneously optimize spatial resolution, imaging speed, signal-to-noise ratio and photodamage^1–3,47–51^. Current technologies remain largely inadequate in coping with (i) the unpredictability of biological events, (ii) biomolecules or phenomena that cannot be easily labeled, and (iii) replicating physiological conditions without perturbation. Overcoming these challenges necessitates a synergistic intersection of hardware, software, and wet lab development. In this Perspective, we discuss some of these key barriers that continue to stymie imaging science. With these current challenges as preambles, we explore how technologies must evolve to advance the various frontiers in life sciences. A complete picture of how life functions can only be attained if we leverage all these technologies together^52^. In essence, we reimagine the future of optical microscopy wherein we can image anything anywhere at any time.

### Anytime

From the movement of single molecules at the millisecond level, through the delicate coordination of cell differentiation over days or weeks, the dynamics of life occur at time scales that span a staggering billion-fold range or more. Light microscopy stands as arguably the only analytical method that can characterize such a wide range of changes in living samples. It is the specimen, however, that will always restrict what can be captured in any single experiment. For instance, following the sub-cellular location of a fluorescent molecule with millisecond precision over several days is, in any practical sense, impossible. There are two important reasons for this: first, any fluorophore can only emit an intrinsically limited number of photons—termed its photon budget^53^. Second, prolonged continuous exposure to intense light will inevitably deteriorate the health of the specimen itself, negating the validity of the very observation being made.

Hence, life scientists have been largely forced to image a specimen either (i) rapidly, using high intensity light, for short amounts of time, or (ii) slowly and/or tolerating less signal to permit longer imaging durations. Worse, this decision must usually be made before the experiment starts— reflecting an often tenuous “best guess” as to the timescales involved. Fortunately, much work over the past decades has been aimed at helping researchers reduce such costly compromises. For example, by confining illumination to the focal plane, light sheet microscopy^7–11^ permits high-speed imaging across large sample volumes, while better preserving sample health and photon budget. This remarkable technique has empowered researchers to, for example, monitor whole-brain neuronal activity in zebrafish by imaging ~100,000 neurons every second at single-cell resolution^10,54,55^. Using suitable reporters, like genetically-encoded calcium and voltage sensors^56,57^, such volumetric and quantitative functional imaging becomes a powerful approach for gaining insights into dynamic biological processes in situ. In tandem, improved chemistry has produced a new generation of bright, genetically encodable dyes capable of withstanding more illumination for longer periods^58^. Further, high-quality information can now be extracted from very low-signal images through judicious use of machine learning techniques^32,59–64^. Yet despite these dazzling advancements, an uncomfortable truth remains: biological systems are replete with rare, transient, and unpredictable events that—while having profound effects—cannot be faithfully captured via time-lapse microscopy.

It is increasingly apparent that “smarter” tools are needed to truly transcend the wide range of biological relevant time scales. Rather than being mere passive observers, imaging systems that reconfigure themselves in response to a specimen present a new set of opportunities^42,65^. Previous developments in adaptive imaging techniques have laid critical groundwork. For example, Stimulated Emission Depletion (STED)^66^ microscopy has benefitted greatly from guided illumination, as was shown by ref. ^67^. In this case, avoiding high power illumination in specific areas of the malaria-causing agent *Plasmodium falciparum* avoided catastrophic sample damage. Moreover, dynamically altering light sheet microscopy alignment to continuously maximize image quality (and therefore optimize illumination power) was instrumental in capturing long-term developmental events in *Drosophila*, Zebrafish, and even mouse embryos with exquisite and unprecedented detail over days of continuous imaging^68,69^.

**Fig. 1:**
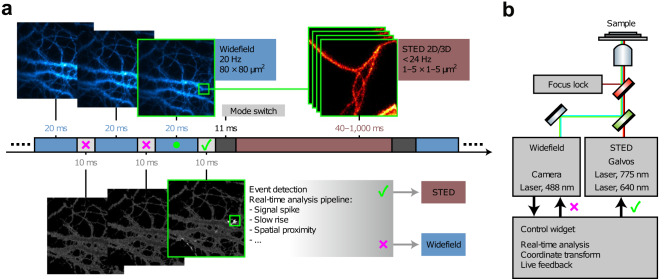
Example of an event-driven microscopy experiment. **a** Scheme of an event-triggered STED (etSTED) experiment. Images from widefield calcium imaging of Oregon Green 488 bAPTA-1 in neurons (blue, top left images) are analyzed by a real-time analysis pipeline (light gray, bottom images). Detection of an event (small green box) triggers modality-switching to STED imaging at the corresponding location (red, top right image stack). **b** Schematic diagram of the etSTED microscope set-up, combining widefield and STED imaging under a control widget. Images are reproduced with permission from ref. ^73^.

Yet, in these cases, the microscopes were completely ignorant to the specific biology they were observing. A more “content aware” methodology is needed to push beyond current barriers to capture other challenging, yet critical processes. Mitochondrial division, for instance, is particularly difficult to characterize with fluorescence microscopy. Individual events typically occur rapidly, sporadically, with high mobility within the cytoplasm, and are notoriously photosensitive. The adaptive illumination approaches outlined above would not ease these challenges. The critical missing ingredient is an “event detector”—a way for the microscope to identify an impending biological process, which can then be used to decide how the instrument should be configured. Recent work by Mahecic et al. ^70^ shows how such a “self-driving” microscope might work. Event-driven microscopy^12,42,70–74^ is not based on a novel optical design or advanced labeling technologies; rather it incorporates a model of biological change, together with a feedback loop. In the case by Mahecic et al., it was assumed that the accumulation of DRP1 protein in mitochondria, accompanied by characteristic shape changes of the organelle itself would indicate a looming fission event. In the absence of these indicators, the microscope adopted a “wait and see” mode—capturing images slowly to preserve photon budget and sample health. During this time, however, each image underwent a sophisticated neural network-based analysis to look for fission indicators. Once found, the microscope automatically switched to a high-speed configuration to capture the division process with high fidelity until it was completed; after which, the system returned to more gentle conditions awaiting the next event. The advantages of this approach were striking. Compared to fixed-rate acquisition, the imaging duration was extended tenfold, allowing five times as many events to be captured while simultaneously reducing photobleaching by the same factor. The same concept can even be used to drive more complex microscope re-configurations. Alvelid et al.^73^ deployed a biological event-driven trigger to switch between relatively gentle epi-fluorescence and illumination-intensive STED imaging modes to better track calcium-mediated endocytosis, as shown in Fig. 1. Multi-modal microscopy^75–78^ is eminently powerful in its own right, but using event-detection to autonomously switch imaging modes will undoubtedly lead to even larger impact. In short, event-driven microscopy stands to provide more biological information for less cost by applying already-developed technologies in a more intelligent manner.

A main hurdle to its widespread adoption, however, lies in the development of robust event detection algorithms. While biologists possess a well-honed ability to predict the onset of a biological process, teaching computers to perform this task accurately and consistently is a non-trivial endeavor. These algorithms rely entirely on the quality and specificity of the training data from which they are derived. Consequently, they are sensitive to both the particular biological problem at hand and the specific microscope used to generate the data. Thus, creating case-specific machine learning models, rather than “out of the box” or “generalized” event detection algorithms, is far more feasible. However, doing so requires easy-to-use tools that simplify this process for non-experts in machine learning. Without this, event-driven microscopy will remain a specialist’s tool, beyond the reach of an overwhelming majority of researchers. Presently, commercial microscopes often incorporate intelligent features, including feedback-driven acquisition. While they do not yet encompass all the advanced features envisioned here, they are engineered for adaptability through user APIs, representing a promising avenue for widespread adoption of event-driven microscopy in the future.

Event-driven microscopy excels at capturing dynamic events in an optimal manner, yet its full potential remains constrained by our ability to label specific molecules or events for observation. Realizing the true power of imaging anytime can only be accomplished if we are able to label anything of interest.

### Anything

The basic constituents of life—proteins, nucleic acids, ions, carbohydrates, and lipids—belie an incomprehensible diversity of biological building blocks. Each is wholly indispensable; yet, optical microscopy has so far been woefully unbalanced in its ability to characterize the labyrinth of interactions between these molecules in living systems. Take, for example, a complex disease such as cancer. The insidiousness of this illness relies on its ability to modify any molecule necessary to ensure its survival and progression^79–81^. By focusing primarily on the proteomic and, to a lesser extent, genetic realms, we put ourselves at a severe disadvantage to even begin to understand the full spectrum of the devastatingly lethal havoc being wreaked in our body, let alone conquer it.

To a large extent, this reflects a difficult biochemical reality: some molecules are simply easier to label than others. The power of fluorescent protein fusions uses the cell’s own machinery to make any gene product visible^4–6^. The more recent revolution brought by CRISPR/Cas9 can even allow such fusions to be expressed from a protein’s genetic locus^82–84^. The same technology can also be used to label specific DNA sequences themselves^85–88^. Even mRNA can be visualized via techniques such as MS2 labeling with single-copy sensitivity^89–92^.

However, other types of biomolecular species have not been afforded such a wealth of available tools, for various reasons posed by the character of the molecule in question. Ions—and in particular metal cations—are unquestionably vital components as well. Yet their small size has thus far defied direct labeling approaches, requiring the use of indirect biosensors. Ca^2+^, and to a lesser extent, Zn^2+^ are two common targets with a bevy of available small-molecule indicators. However, their specificity has been questioned, and long-term toxicity remains a concern^93,94^. Moreover, they cannot be targeted to specific cell populations. Fortunately, it is now possible to use genetically encoded fluorescent proteins to image ionic species^95–98^. GCaMP and its variants have become indispensable tools for live-cell Ca^2+^ imaging^56,99,100^. Similar tools for visualizing Zn^2+^, pH, biomechanical forces, and voltage gradients are also available^57,93,101–105^. But the palette of genetically encoded ion sensors is still limited. Other vitally important species such as Na^+^, Mg^2+^, Fe^2+^, and others still await robust, reversible, and targetable sensors to render these vital ions visible. To do so requires the full power and ingenuity of the protein engineering field and beyond.

**Fig. 2:**
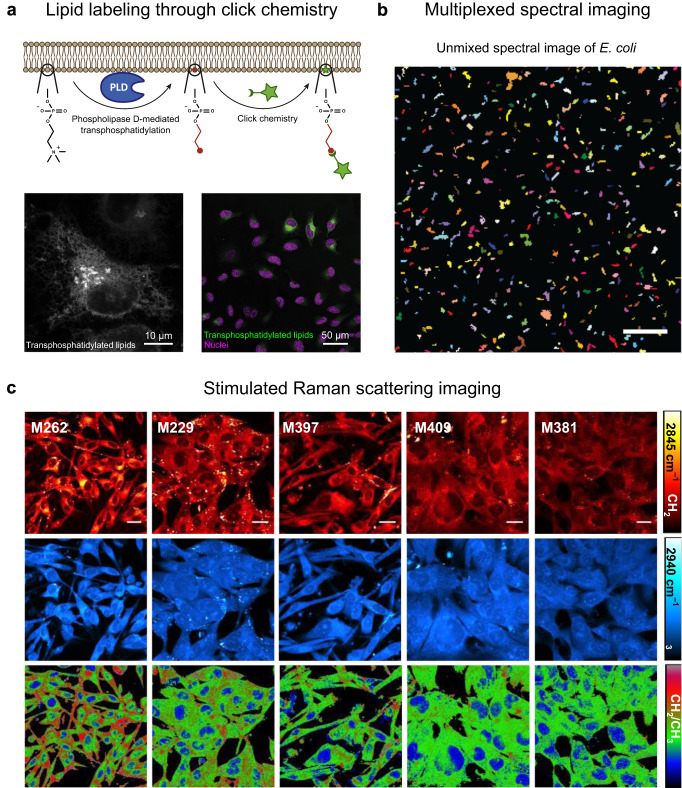
Examples of currently available tools that, if integrated with other methods, could potentially pave the way towards the goal of imaging “everything”. **a** Target lipids that are transphosphatidylated by phospholipase D (PLD) can be visualized using click chemistry. The bottom images show labeled sites of PLD activity in HeLa cells. Scale bar = 10 µm (bottom left image), 50 µm (bottom right image). **b** Unmixed spectral image of an artificial mixture of 120 differently labeled *E. coli*. Scale bar = 25 µm. **c** Representative stimulated Raman scattering images of five melanoma cell lines (showing decreased differentiation from left to right) corresponding to the lipid peak (2,845 cm^−1^; top row in red), protein peak (2940 cm^−1^; middle row in blue), and the ratio of lipid/protein (bottom row). Scale bar = 20 µm. The images in (**a**–**c**) are reproduced with permission from refs. ^113,124,136^, respectively.

Lipids and carbohydrates, however, are arguably the most inaccessible “dark matter”^106^ in biology. The plasma membrane alone contains hundreds of distinct lipid types^107^, with various chemical modifications possible for many of them. Even more troubling, glycans^108–110^ represent the most abundant biomaterial on earth. It is ironic then, that targeting a specific variety of lipid or glycan for imaging remains a highly underdeveloped field. To date, two general approaches have been explored. The first uses a fluorescent lipid- or glyco-binding protein to infer the location of the target—provided there exists a suitably specific and sensitive protein available^111–113^. Yet this can only offer an indirect measure of content and function. Bioorthogonal or “click” chemistry^113–116^ can be used to synthesize a fluorescent lipid or carbohydrate within the cell (Fig. 2a). This method, while perhaps more elegant, requires significant chemical expertise to undertake. Despite several available commercial tools, click chemistry has not been as widely adopted as other labeling techniques.

To be sure, developing more robust, broadly applicable, and most importantly, accessible labeling strategies for the full range of biomolecules is critical to the future of live cell imaging. But a more fundamental question looms. Being able to image anything naturally leads one to wonder— could we image everything? And should we? Much of biology relies on a reductionist approach whereby only a handful of molecular players are investigated at a time; this will undoubtedly remain a bedrock of bioscience. Yet bringing a systems-level approach to live imaging offers tantalizing possibilities. It is also a challenge of monumental proportions that resists a single “catch all” solution. The dream of simultaneously imaging the behavior of every molecular player in a signaling cascade may sound far-fetched. But, our current capabilities, if integrated in novel ways, may make this goal achievable. Spectrally-resolved imaging can increase the number of simultaneously detectable fluorophores 4- to 5-fold, provided a robust unmixing algorithm can be employed^117–119^. Further, we can look to other readouts besides fluorescence intensity and color. Fluorescence lifetime^120–122^ or photobleaching rates^123^ provides orthogonal imaging modes that can further increase the number of distinguishable biomolecules in an image.

However, systems-level live microscopy necessitates more than just better microscopes. Detecting *ca*. 100 distinct molecules (or more) requires even more ingenuity, as the number of suitable fluorophores (and our ability to introduce them in a sample) quickly becomes exhausted. “Bar code” labeling—where molecules are labeled with a unique ratio of multiple fluorophores—takes advantage of combinatorics to create effectively hundreds or even thousands of unique fluorescent signatures, which when combined with suitable analysis, can begin to reach systems-level analysis^124^ (Fig. 2b). Using bar-code imaging with chemigenetic tools such as halo, SNAP, and CLIP handles^125,126^ in living samples may make it possible to accomplish this with genetic specificity in live systems.

The use of fluorescent labels, however, comes with important considerations, as they may perturb, often in pernicious ways, native biological processes^127–129^. Data interpretation can be skewed by artifacts arising from fluorophore-fluorophore interactions, their susceptibility to the local microenvironment, and phototoxicity, among many other effects^51,127,130,131^. Consequently, it becomes essential to design experiments meticulously, including the use of proper controls, and to evaluate the imaging data with a critical eye. Effective checks include assessing whether the labeled samples maintain normal morphology and behavior, and whether consistent results are obtained by using different tags. In certain scenarios, the intrinsic optical characteristics of biological molecules can be leveraged for label-free imaging^26,29^. Quantitative phase imaging^132,133^, stimulated Raman microscopy^134–136^ (Fig. 2c), as well as second harmonic generation and other orthogonal imaging techniques^25–28^ can all be brought in to supplement and complement multiplexed fluorescence microscopy.

In short, the technologies needed to image anything—and perhaps even “everything”, may be in reach sooner than we think. However, crossing the finish line requires that chemical tool developers, optical engineers, and biologists work in even greater synchrony toward common goals. That being said, the size and complexity of such multidimensional data will surely overwhelm our current capacity to handle and analyze them. The dimensional reduction and analysis techniques already developed for bulk genetic and proteomic studies must be adapted to preserve the spatiotemporal resolution that only imaging can provide.

The combined capabilities to image at anytime and study anything will undoubtedly accelerate groundbreaking advancements in biological research. However, they will remain limited in their usefulness unless we overcome another significant obstacle—the ability to image anywhere.

### Anywhere

While expanded and multiplexed molecular labeling technologies and “smart” microscopes will be critical to the future of imaging science, there remains the considerable challenge of applying these technologies anywhere within in a complex biological system. Many of the critical processes of life can only take place within a living organism under physiological conditions that cannot be replicated by single-cell models, or even in tissuemimetic systems such as organoids^137–139^. However, illumination light and signals of interest must be delivered to and detected from deep within an organism to faithfully image such processes. To gain sufficient penetration depth often requires invasive procedures, which can threaten the wellbeing of the specimen. These compounding difficulties can quickly become insurmountable with current technologies, thus restricting biologists from visualizing processes in their most relevant physiological contexts.

**Fig. 3:**
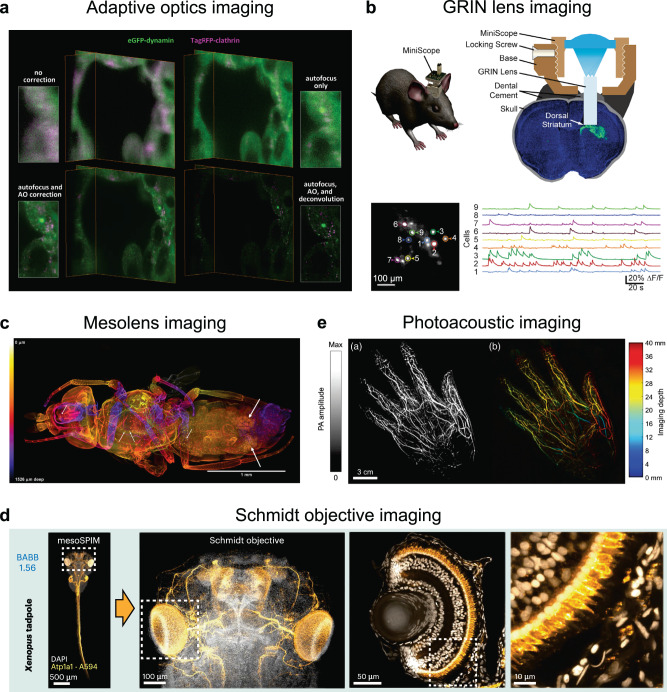
Examples of different available technologies to image biological samples with greater depth and/or larger FOV. **a** Comparison of different corrections, including the use of adaptive optics, on images of a live human stem cell-derived organoid expressing dynamin and clathrin obtained using a lattice light sheet microscope. **b** The top image shows a schematic of a MiniScope equipped with a GRIN lens mounted on a mouse’s head. The bottom image shows a representative fluorescent image of medium spiny neurons labeled with GCaMP6s. The traces indicate calcium transients from ROIs 1–9. Scale bar = 100 µm. **c** Projected image of the entire volume of a female *Drosophila* obtained using a Mesolens. Scale bar = 1 mm. **d** Benzyl alcohol/benzyl benzoate (BABB)-cleared *Xenopus tropicalis* tadpole stained for Atp1a1 (Alexa Fluor 594, orange) and nuclei (DAPI, grayscale). The sample was first imaged on a mesoSPIM light sheet microscope, and then using a Schmidt objective. The large Schmidt FOV allows imaging of both the entire head (~800 μm across) and individual developing photoreceptors in the eye. Scale bar = 500 µm, 100 µm, 50 µm, and 10 µm, respectively, for images going left to right. **e** Photoacoustic images of a 3-D reconstructed maximum amplitude projection and corresponding color-encoded depth-resolved image of a volunteer’s right hand. The blood vessel network can be clearly visualized. Scale bar = 3 cm. The images in (**a**–**e**) are reproduced with permission from refs. ^38,154,157,158,178^, respectively.

To be able to follow biological processes at-will means that an imaging system must contend with the optical properties of the specimen, as well as maximizing imaging depth and field of view (FOV). What fuels our motivation to image anywhere within a biological system is to explore its vast complexity in context. Yet, it is this very same complexity that distorts our view and misleads our interpretation. When imaging through heterogenous tissue, light is inevitably scattered, absorbed, or aberrated^140^. In recent years, the correction of optical aberrations has become reasonably achievable through adaptive optics (AO). By measuring the distortion of the optical wavefront propagating in and out of a specimen, a deformable mirror or other optic can apply the inverse distortion and restore image quality. The technology has been successfully demonstrated^38–41^ (Fig. 3a), yet remains largely inaccessible to the broader biological community. Before AO can be implemented universally, a more user-friendly or “one-click” interface must be adopted. Contrary to common assumption, AO is only useful in correcting *aberrations*—it cannot correct image degradation due to scattered or absorbed light. Unless a sample is truly transparent, increasing amounts of excitation and emission light will be scattered or absorbed by the specimen with increasing imaging depth. This has placed a fundamental limitation on where we can observe dynamic processes within a complex sample. Working with optically transparent samples such as chemically cleared specimens^141,142^, *Danio Rerio*, or *Caenorhabditis Elegans* can mitigate this issue even for something as big as a human embryo^143^. Ultimately, however, the holy grail is to visualize live specimens with sufficient resolution at any depth. Even commonly used intravital imaging techniques, like multiphoton microscopy^144,145^, limits accessibility to a few hundred microns. Imaging in the NIR II window (1000–1700 nm) shows promise for deeper tissue penetration (mm range) due to reduced light absorption, scattering, and lower autofluorescence. However, its widespread adoption hinges on multiple technical advancements, including better fluorophores and suitable detectors^146–148^. Additionally, signals that have been cloaked by optical aberrations from deep tissue imaging can now be partially recovered using software tools. Machine learning-based approaches^31,32^ can accomplish this if appropriate training data is provided. However, the validation and reproducibility of such models remains a challenge. Even so, no true single solution such as AO exists for reducing the deleterious effects of light scattering on an image—such a development would fundamentally reshape how live microscopy is performed.

Further compounding the challenges posed by the sample, the objective lens working distance places an unassailable limit on the imaging depth. Working distance scales inversely with numerical aperture (NA), forcing a compromise between depth and resolution. One way to sidestep working distance is to eliminate it altogether. Rather than trying in vain to collect photons beyond the working distance, a Gradient-Index (GRIN) lens^149^ can be implanted into a specimen to relay photons from a deeper portion of the sample back to the focal plane of the objective. Likewise, a GRIN lens can also be affixed to an optical fiber to create an endomicroscope^150–154^ (Fig. 3b). This allows researchers to effectively image any location they can access with a small, flexible arm, which is especially powerful for accessing small canals. However, imaging at any location is hardly equivalent to imaging anywhere. The limited GRIN lens FOV immediately pigeonholes the biologist to a preselected, restricted region, blinding the observer to the broader, interrelated biological landscape. One additional, and rarely appreciated, complication is that such highly invasive procedures on the unfortunate specimen could have unintended, detrimental, and unpredictable biological consequences. A localized inflammatory response triggered by the insertion of the GRIN lens could be misattributed as the involvement of the immune system in the very biological event being observed^155^. Worse yet, such reactions may trigger other unrelated, secondary events that misguide data interpretation.

Drawing conclusions from data lacking in context is inherently prone to gross misinterpretation. In microscopy, context is in many ways closely linked to FOV. Unfortunately, FOV and resolution are fundamentally at odds, forcing microscopists to compromise between breadth and specificity. Circumventing FOV limitations can be readily achieved by image tiling, a commonly available feature. Yet, this approach suffers from poorer imaging speed and image stitching artifacts. A true solution requires a complete reimagination of objective lens design, as is exemplified by the Mesolens^156^. Combining a large (6 mm) FOV with a comparatively high NA (0.5) and a long working distance (3 mm), the Mesolens can capture an entire adult *Drosophila* with sub-cellular resolution^157^ (Fig. 3c), with a commercial version available in the form of the two-photon random access mesoscope (2p-RAM). Another recent solution is the Schmidt objective^158^ (Fig. 3d), inspired by the Schmidt telescope, that replaces lenses with a spherical mirror and a refractive correction plate. Such a radical redesign of a microscope objective endows it with a highly desirable combination of advantages—high NA (0.69-1.08), large FOV (1.1–1.7 mm), long working distance (11 mm), while being compatible with a wide range of sample immersion media. However, FOV is not a strictly two-dimensional concept. Collecting a canonical “z stack” is akin to 2D tiling for an increased FOV: multiplicatively slower, inversely related to resolution, and increasingly prone to photodamage and motion artifacts. A single-shot approach to collecting an entire 3D volume alleviates these burdens. This has been realized on small length scales through multifocal microscopes^159,160^ and large length scales through light field microscopes^161–163^. Recently, light field microscopy was paired with a mesoscopic objective lens for a lateral and axial FOV of 4 and 0.2 mm, respectively. Because of its single snapshot 3D capability, neural activity in a mouse cortex could be imaged at an astounding 18 volumes per second over this large FOV—a markedly impressive breakthrough in rapid 3D imaging^164^. An immediate challenge, however, is the sheer scale of large FOV, volumetric, live microscopy data. The logistical headaches for data storage, transfer, and analysis are considerable^165–169^. This burden can be somewhat ameliorated via lossless compression methods^170^ to reduce data size and/or adaptive microscopy techniques that can reduce collection of non-informative data. However, the challenges of big data extend beyond storage. Data processing and analysis requires access to powerful computational resources, appropriate software, and technical expertise to derive meaningful insights from the data^32,165,169,171,172^. The adoption of automated analysis workflows is imperative because manual analysis is impractical at such scales due to its laborious nature and susceptibility to human bias and error, and it is crucial to properly train machine learning models for this purpose. Given these substantial challenges, biologists no longer have the luxury to treat data handling and processing as an afterthought, and must proactively prepare and ensure all essential resources are in place before even commencing their experiments.

Equally worrisome are the challenges of applying large FOV volumetric imaging deep within a living organism. At these substantial depths, the spatial context of the surrounding tissue cannot be ignored, yet our current solutions for large FOV imaging still necessitate the invasive procedures previously described. Imaging the surrounding, native environment is effectively pointless if this very tissue must be destroyed simply for access. We can draw inspiration from clinical imaging to achieve this.

One clinically accepted, ethical way to peek at an unborn fetus is through ultrasound imaging^173^. It is the method of choice precisely because ultrasound is not encumbered by the limitations of photodamage and invasive approaches in conventional optical imaging. Furthermore, it routinely achieves centimeters scale imaging depth. Conversely, ultrasound lacks the molecular specificity and resolution that light microscopy routinely provides. The two imaging modalities, however, can complement each other synergistically if properly integrated. Specific molecules can be excited by certain wavelengths of light and the ultrasound originating from its thermal vibration can then be detected, forming the basis of photoacoustic imaging^174–177^. Many molecules emit signature photoacoustic signals, and thus can act as intrinsic contrast agents, enabling label-free imaging. A popular example is hemoglobin, which exhibits wavelength-specific photoacoustic effects in oxygenated and deoxygenated states, and is widely used for imaging tissue vascularization and quantification of tissue oxygen consumption^177–180^ (Fig. 3e). For cases when molecules of interest do not provide sufficient photoacoustic contrast, specificity can be introduced into the sample through exogenous contrast agents like gold nanoparticles and fluorescent dyes^181^. Another agent which provides specificity and multiplexing for photoacoustic imaging, similar to fluorescent proteins, are gas vesicles^182^. Initially identified in aquatic microbes as regulators of cellular buoyancy, these gas-filled protein nanostructures produce strong ultrasound contrast and can be tuned to collapse at different frequencies. They have even been utilized as genetically-encoded reporters^183^. Various other techniques^176,184,185^ such as magnetic resonance imaging^186–188^, computed tomography^189,190^, positron emission tomography imaging^191,192^, optical coherence tomography (OCT)^193,194^, bioluminescence imaging^195–197^, hyperspectral imaging^117–119^, etc., have also helped make significant progress in imaging biological processes in their native tissue environment. The natural progression is then to consider the entire environment surrounding the model organism itself.

Beyond the optical challenges of imaging deep into a complex specimen are the difficulties imaging samples that are themselves moving. Specimens often grow or move out of a FOV during a prolonged imaging experiment. Recent years have seen integrated software pipelines capable of monitoring and adjusting the sample position, which is specifically useful for developing embryos^69^. However, the future of imaging must include approaches to mitigate the movement of even more complex specimens. Intravital imaging^198,199^ is exceptionally powerful for imaging within living vertebrates such as mice or rats. By opening a window to the brain, kidneys, or other organs, one can visualize dynamic processes live within the animal^200^. However, the animal is often restrained and/or heavily sedated, which may alter the biological processes being studied. Systems capable of performing intravital imaging on animals free to walk, eat, and perform other normal functions are critical^201–206^; this requires, however, an overhaul of hardware, software, and wet lab tools. More fundamentally, this necessitates a shift in thinking from bringing the sample to the microscope to adapting the microscope to the sample.

Such a profound change in perspective makes it evident that the next logical step is to extend the notion of imaging anywhere to the environment in which the microscopy is being performed. Quantitative, research-grade microscopy is almost exclusively performed in laboratory spaces, requiring the specimen to be brought from the field to the microscope. This inherently, and often severely, restricts the problems that can be tackled with microscopy. This is especially the case regarding infectious disease research. In many pressing cases where microscopy can make an immediate impact on dire health crises, limited windows for sample viability and biosafety concerns will often restrict specimen transport and examination in a laboratory space. Therefore, it is imperative that the future of imaging consider the concept of moving the microscope to the biology, where the microscope is no longer seen as a static instrument but rather as a flexible tool adaptable to the diverse conditions of the field. For instance, the LoaScope^207^, which has been used to rapidly detect *Loa loa* microfilariae in peripheral blood, has successfully guided the treatment strategy for thousands of infected patients in Central Africa^208^. Many efforts among microscopy developers aspire towards gaining the highest spatial resolution, deepest penetration depth, or fastest imaging speed; while this is laudable, human health can often be better served by adapting current technologies to readily, and robustly, function in challenging point-of-care environments and other settings that do not lend themselves easily to microscopy. Some field microscopes, such as the Em1 portable microscope, are commercially available, although higher costs and reduced configurability may limit their applicability for some users. Open-source alternatives, like the Octopi microscope^209^, offer significant advantages in this regard as they can be more flexible for particular needs and are often more budget-friendly.

### Outlook

Ironically, the ability to image multiple biological processes in action within a large volume of biological samples will create more problems than it can answer, as data “overload” will get more severe as imaging technologies progress. It will inevitably supersede the human capability to process and comprehend the information. In fact, it may even overwhelm the ability of artificial intelligence to tackle the challenge as its capacity is limited by how the system is trained. New biological theories that can potentially guide such effective and comprehensive training are yet to be fully developed, so the confusion will likely worsen before it gets better.

As much as we have advocated the importance of testable hypothesis in guiding microscopy-based experimental design^53^, in this case it may be a liability that would limit one’s ability to see beyond what is dictated by the hypothesis. This rigidity may blind the observer from potentially new discoveries hidden in the complex interplay of biological process – the raison d’etre of multi-dimensional imaging. Conversely, the complexity of modern microscopy datasets, while offering a vast landscape for exploration, can also quickly devolve into a labyrinth of spurious relationships and biased postulations unless they are further challenged by subsequent experiments. More importantly, such large-scale exploration may not be effectively accomplished by a single group. This argues for the importance of community-driven data mining, where the data can be viewed from multiple perspectives by a wide range of expertise. Such efforts have been actively ongoing for volumetric electron microscopy but remains at its infancy for optical imaging.

**Fig. 4:**
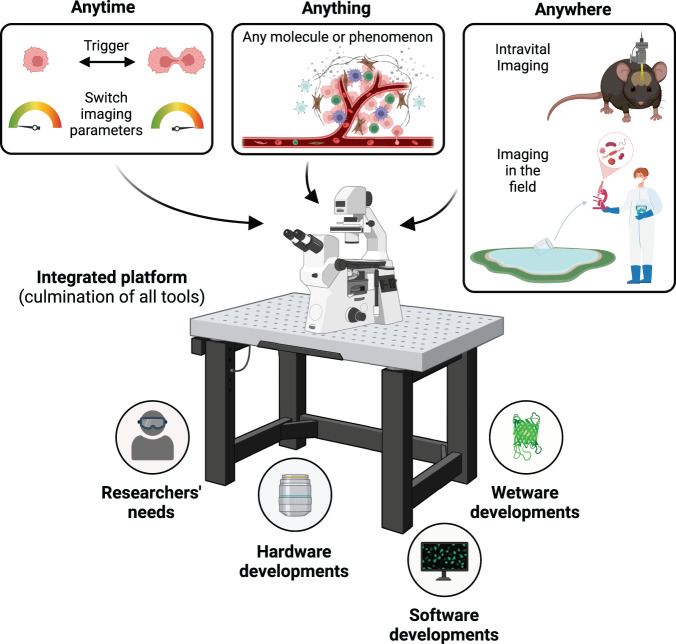
A vision of the future of live imaging. A culmination of various tool developments, integrated with easy and equitable access to these technologies, will enable imaging anytime, anything and anywhere, thereby powering biological breakthroughs.

The future of our ability to visualize the processes of life in the context of living specimens is one that is both exciting and challenging. As we push the boundaries of live imaging, it is clear that current microscopy technologies cannot provide a complete picture by themselves. The solutions to these obstacles will necessarily come from the collective, interdisciplinary efforts^52^ of microscope builders, software specialists, and probe developers to develop new transformative technologies (Fig. 4). However, often forgotten or ignored in the development process is the end user—bioscientists who rely on these tools to navigate through the challenges inherent to biological research. Failing to keep their needs at the forefront risks isolating the target audience, thereby resulting in a poor outcome given the expenditure and efforts put into developing the technologies. To truly revolutionize the field of live cell imaging, this chasm between tool creation and its widespread adoption must be bridged. The involvement of the end users in the development process, and incorporating their input and feedback, is indispensable.

In this paper, we consider the ability to perform imaging experiments “anywhere” to also include wider adoptability of the microscopy technology, especially in regions where accessibility to technologies and infrastructure is a challenge. The workflow of microscopy technology development cannot be considered complete upon publication of the technology but must be extended to include the necessary strategies to bring the newly developed tools into the hands of scientists who need them. This is an under-appreciated challenge of technology development, often overlooked even by funding organizations that support development of the very technology in question. Turning a blind eye to technology dissemination creates a missed opportunity for the scientific community to reap the utmost transformative power from the technology, and an even bigger loss for funders to maximize the impact-per-dollar of their investment. This clear demarcation of responsibility, in which technology developers do not see themselves as champions of their own inventions, is often tacitly endorsed by funders. In fact, many funders do not emphasize dissemination strategies as part of the success metrics of technology development. More importantly, to ignore dissemination is antithetical to the very spirit of technology development, for it channels the developer’s attention and effort away from taking the product past the finishing line where the technology can be widely usable. This has further precipitated a situation wherein academic research is saddled with potentially transformative technologies, rarely progressing beyond the proof-of-concept stage. The rare instances of successful and widespread commercialization, such as the lattice light sheet^74^, multiview light sheet^69^, swept confocally-aligned planar excitation^210^, and chip-based microscopes^211^, occurred precisely because of the active efforts of the developers to disseminate these technologies. Therefore, it is essential for all stakeholders, including researchers, funders, and technology developers, to foster an ecosystem of innovation that supports the entire lifecycle of technology development—from ideation to dissemination. However, commercialization is not the sole demonstration or proof of successful dissemination. While partnerships with industry to facilitate the distribution of new technologies is a positive step, the process of commercialization can take several years and the products may not be affordable to a broad range of researchers. Therefore, it is imperative, in parallel, to develop other avenues of technology dissemination via open-source tool development^212^, training and education programs^213,214^, and creation of open-access centers^165^. The emergence of affordable, open-source research-grade microscopes represents a rapidly advancing area, with innovations like the openFrame^215^ microscope and Flamingo microscope^216^ effectively lowering entry barriers and offering a modular design for convenient upgrades^217^. Similarly, open science initiatives for sharing of research reagents, such as Addgene plasmids^218^ and JaneliaFluor dye distribution program^219^, must be embraced and are necessary for the advancement of scientific knowledge and collaboration.
